# Isoprostanes and Neuroprostanes as Biomarkers of Oxidative Stress in Neurodegenerative Diseases

**DOI:** 10.1155/2014/572491

**Published:** 2014-04-29

**Authors:** Elżbieta Miller, Agnieszka Morel, Luciano Saso, Joanna Saluk

**Affiliations:** ^1^Department of Physical Medicine, Medical University of Lodz, Hallera 1, Lodz, Poland; ^2^Neurorehabilitation Ward, III General Hospital in Lodz, Milionowa 14, Lodz, Poland; ^3^Department of General Biochemistry, Faculty of Biology and Environmental Protection, University of Lodz, Pomorska 141/143, 90-236 Lodz, Poland; ^4^Department of Physiology and Pharmacology “Vittorio Erspamer”, Sapienza University of Rome, Rome, Italy; ^5^Department of Toxicology, Faculty of Pharmacy with Division of Medical Analytics, Wroclaw Medical University, Borowska 211, 50-556 Wroclaw, Poland

## Abstract

Accumulating data shows that oxidative stress plays a crucial role in neurodegenerative disorders. The literature data indicate that *in vivo* or postmortem cerebrospinal fluid and brain tissue levels of F_2_-isoprostanes (F_2_-IsoPs) especially F_4_-neuroprotanes (F_4_-NPs) are significantly increased in some neurodegenerative diseases: multiple sclerosis, Alzheimer's disease, Huntington's disease, and Creutzfeldt-Jakob disease. Central nervous system is the most metabolically active organ of the body characterized by high requirement for oxygen and relatively low antioxidative activity, what makes neurons and glia highly susceptible to destruction by reactive oxygen/nitrogen species and neurodegeneration. The discovery of F_2_-IsoPs and F_4_-NPs as markers of lipid peroxidation caused by the free radicals has opened up new areas of investigation regarding the role of oxidative stress in the pathogenesis of human neurodegenerative diseases. This review focuses on the relationship between F_2_-IsoPs and F_4_-NPs as biomarkers of oxidative stress and neurodegenerative diseases. We summarize the knowledge of these novel biomarkers of oxidative stress and the advantages of monitoring their formation to better define the involvement of oxidative stress in neurological diseases.

## 1. Introduction

The CNS (central nervous system) is very vulnerable to oxidative injury due to its high oxygen demand, high level of polyunsaturated fatty acids (PUFAs), and weak antioxidant defenses. The vulnerability of the brain to oxidative damage increases with the age due to reduced integrity of the blood-brain barrier (BBB) and increased mitochondrial dysfunction [1–19]. Brain aging and neurodegeneration are characterized by chronic inflammation with persistent microglial activation and higher level of proinflammatory cytokines [4]. In addition, it promotes oxidative stress and neuronal damage. Neurons are particularly vulnerable to oxidative damage not only due to excitotoxicity but also to mitochondrial dysfunction. Moreover, neuronal membranes have plenty of unsaturated fatty acids. At higher concentrations, reactive oxygen/nitrogen species (ROS/RNS) cause neural membrane damage. Therefore, it can change not only membrane fluidity but also decreased activities of membrane-bound enzymes, ion channels, and receptors. The main sources of ROS/RNS are the mitochondrial respiratory chain, an uncontrolled arachidonic acid (AA) cascade, and NADPH oxidase (nicotinamide adenine dinucleotide phosphate-oxidase) [5, 6].

It is known that both inflammation and oxidative stress contribute to the development of various neuropathologies including Alzheimer's disease (AD), Parkinson's disease (PD), and multiple sclerosis (MS) [5, 6, 20]. As discussed by Guest et al. [7] the cerebrospinal fluid (CSF) of participants aged over 45 years contained statistically higher amounts of the oxidative damage marker F_2_-isoprostane (F_2_-IsoPs) and the inflammatory cytokine IL-6.

Brain response to oxidative stress-mediated neurodegeneration is very complex. All brain structures are involved in this multifactorial process. Astrocytes constitute approximately 90% of human brain and protect neurons from excitotoxicity through glutamate uptake system, and on the other side astrocytes contribute to the extracellular glutamate* via* reversed glutamate transporter [5]. Moreover, they may undergo astrocytosis after dopaminergic cell loss and are involved in the inflammatory processes. In general, inflammation is a protective response. The main mediators of neuroinflammation are microglial cells. Microglial cells consist mainly of macrophages and react to oxidative stress by transformation into activated microglia that are characterized by amoeboid morphology and rapid migration. Chronic activation of microglia may cause neuronal damage through the release of potentially toxic molecules such as proinflammatory cytokines, matrix metalloproteinases, ROS/RNS, proteinases, prostaglandin E_2_, complement proteins, and growth factors and also leads to the DNA and RNA damage [5, 21]. These factors have neuroprotective properties but on the other hand they can be responsible for acceleration of oxidative stress and neurodegeneration.

Markers of lipid peroxidation include different molecules such as 4-hydroxy-trans-2-nonenal (4-HNE), 4-oxo-trans-2-nonenal (4-ONE), acrolein, isoprostanes, and isofurans. These markers are derived from AA, which is released from neural membrane glycerophospholipids through the activation of cytosolic phospholipases A_2_ (cPLA_2_) [22].

Lipid peroxidation is a hallmark of oxidative stress. High level of lipid peroxidation products is a characteristic for many human diseases, especially neurodegenerative diseases. It can cause damage to cellular membranes through changes of membrane organization and alteration of membrane integrity, fluidity, and permeability [23].

## 2. Biomarkers

Biomarkers are defined as the indicators of normal biological processes or pathologic processes that can be objectively measured and evaluated [1]. The well-characterized, appropriate biomarkers may be used for health examination, diagnosis of pathologic processes at early stage, assessment of treatment response, and prognosis. Noninvasive measurements of circulating levels of specific biomarker are useful along the whole spectrum of the disease process and before diagnosis biomarkers could be used for screening and risk assessment of the diseases [2]. Moreover, biomarkers may be used for precise measurement of oxidative stress status* in vivo* [3]. Among the biological molecules, lipids appear to be the most susceptible to the attack of ROS/RNS [24, 25] and lipid peroxidation has been implicated in the neurodegeneration [11]. Therefore, the levels of lipid peroxidation products may be used as a biomarker for the measurement of oxidative stress status* in vivo* in neurodegenerative diseases [3]. The levels of lipid peroxidation products in biological fluids and tissues of human subjects have been measured extensively [26]. Presently, various lipid peroxidation products are applied for assessment of lipid peroxidation and oxidative stress status* in vivo.*

The measurement of F_2_-IsoPs is currently the best available biomarker of lipid peroxidation [8, 10, 22, 24].

## 3. The Isoprostanes Pathways

In the mid-1970s, it was shown that PG-like compounds could be formed* in vitro* by the nonenzymatic peroxidation of purified PUFAs. F_2_-IsoPs have been discovered in 1990 by Milne et al. [27] and Roberts II and Morrow [28] and since then they collected a lot of evidence that these compounds might be biomarkers of lipid peroxidation and oxidative stress* in vivo* as well as* in vitro*.

F_2_-IsoPs are a unique prostaglandin-like products, which are formed* via* nonenzymatic, free radical-mediated peroxidation of polyunsaturated fatty acids—for example, AA [28, 29]. The oxidation of AA proceeds by many competing reactions to give numerous products. IsoPs containing a variety of prostane ring structures are composed of various isomers including F_2_-IsoPs, which are isomeric to PGF_2*α*_ [27, 30, 31] and D_2_/E_2_-IsoPs, which are isomers of PGD_2_ and PGE_2_, respectively [32]. The mechanism of F_2_-IsoPs formation involves several steps. In the first stage, ROS reacts with the arachidonic acid and undergoes abstraction of an bisallylic hydrogen atom to yield an arachidonyl carbon-centered radical. What is more, there is insertion of oxygen, which leads to the formation of peroxyl radicals. Four different peroxyl radical isomers are formed depending on site of hydrogen abstraction and oxygen insertion. Peroxyl radicals isomers undergo 5-endocyclization and a second molecule of oxygen adds to the backbone of the compound to form four bicyclic endoperoxide intermediate regioisomers—PGH_2_-like compounds. These unstable bicycloendoperoxide intermediates are reduced to the F_2_-IsoPs and four F_2_-IsoPs regioisomers are formed [30, 33, 34]. This regioisomers are reduced to four series of F-ring regioisomers (15-, 8-, 12-, 5-series), each consisting of eight racemic diastereoisomers. These regioisomers are depending on the carbon atom to which the side chain hydroxyl is attached [8].

To sum up, the biosynthetic steps of IsoPs include formation of the following:

- three arachidonyl radicals,
- four peroxyl radical isomers with subsequent endocyclization; finally, formation of bicycloendoperoxide regioisomers, which are reduced to F_2_-IsoPs; IsoPs are compounds that have F-type prostane rings isomeric to PGF_2*α*_.

The alternative ring structures, D/E-type and A/J-type prostane, are formed by the same mechanisms [32, 34]. So that E- or D-ring and thromboxane-ring compounds of IsoPs are formed during the rearrangement of isoprostane endoperoxides* in vivo*. E_2_- and D_2_-IsoPs are not terminal products of the IsoP pathway. These compounds are unstable and readily undergo dehydration* in vivo* to yield A_2_/J_2_-IsoPs. The cyclopentenone IsoPs might be neurotoxic products of the IsoPs pathway and might contribute to the pathogenesis of oxidative neurodegeneration. A_2_-/J_2_-IsoPs contain *α*,*β*-unsaturated carbonyls, which rapidly adduct cellular thiols and these cyclopentenone IsoPs induce neuronal apoptosis and promote neurodegeneration [27].

The other electrophilic lipid peroxidation products can also damage neurons. The *γ*-ketoaldehydes (e.g., isoketals, isolevuglandins), highly reactive acyclic compounds, might be formed as a products of IsoPs endoperoxide rearrangement [35].

PUFAs are the most susceptible to free radical attack and, in general, oxidizability increases as the number of double bonds increases. So, the oxidizability of PUFAs can be estimated by the linear increase in the rate of oxidation with the increasing number of active methylene groups located between two bonds. From such correlation, the oxidizability of each PUFA is increased for about twofold for each active methylene group. Thus, the oxidizability of common fatty acids is as follows: linoleic acid (18 : 2) < arachidonic acid (20 : 4, *n* − 6) < eicosapentaenoic acid (EPA, 20 : 5, *n* − 3) < docosahexaenoic acid (DHA, 22 : 6, *n* − 3) [27, 36].

The oxidation mechanisms of IsoPs are well known, but they are not the only substrate for the IsoPs pathway. The presence of at least three double bonds in fatty acid molecule allows the cyclization.

F2-dihomo-isoprostanes (F2-dihomo-IsoPs) are the peroxidation products from adrenic acid, which is the main component of myelin. The great amount of DHA is observed in brain but primarily found in white matter and is associated with myelin. White matter is commonly damaged by ischemic stroke and is uniformly damaged in MS. F2-dihomo-IsoPs are generated in significant amounts from adrenic acid and their levels are greatly increased in settings of oxidative stress occurring in the white matter portion of the human brains. Roberts II and Milne [8] demonstrate that, proportionally, levels of F2-dihomo-IsoPs in white matter undergoing oxidative injury increase to a greater extent than IsoPs and NeuroPs derived from AA and DHA, respectively. Their studies suggest that the quantification of F2-dihomo-IsoPs might be a selective marker of white matter injury* in vivo* [8].

F2-dihomo-IsoPs are also present in kidney, adrenal glands, and tissues and might be regarded as an early marker of lipid peroxidation in Rett syndrome—a disorder of the nervous system that leads to developmental reversals, especially in the areas of expressive language and hand use [37].

### 3.1. AA Is Not the Only One PUFA That Can Be Oxidized to Form IsoPs

By the peroxidation of the *ω*-3 PUFA, EPA and DHA, F-ring IsoPs have been generated. The IsoPs-like compounds generated from this acid are named NeuroPs [8].

F_3_-IsoPs are formed in abundance* in vitro* and* in vivo* from EPA nonenzymatically peroxidation [38–40], while DHA may be oxidized nonenzymatically into F_4_-, D_4_-, E_4_-, A_4_-, and J_4_-neuroprostanes (F_4_-, D_4_-, E_4_-, A_4_-, and J_4_-NeuroPs) [38, 39]. AA is relatively evenly distributed in brain with similar concentrations in gray matter and white matter, and within glia and neurons. Unlike AA, DHA is highly concentrated in neuronal membranes to the exclusion of other cell types. Moreover, F_4_-NeuroPs are by far the most abundant products of this pathway in the brain [32]. The quantification of F_4_-NeuroPs provides a highly selective quantitative window for neuronal oxidative damage* in vivo.* Thus, F_2_-IsoPs quantification is a reflection of oxidative damage to the brain in general and F_4_-NeuroPs in particular [40, 41]. Roberts II and Milne [8] have found that the level of IsoPs produced from the oxidation of EPA significantly exceeds those of the F_2_-IsoPs generated from AA. This is because EPA contains more double bonds, and therefore, it is more easily oxidizable. The authors have also observed that EPA supplementation markedly reduced levels of arachidonate-delivered F_2_-IsoPs mouse heart tissues by over 60%. Such observations are crucial because F_2_-IsoPs are generally considered as a proinflammatory molecules associated with the pathophysiological sequelae of oxidant stress. It is thus surprising to propose that the part of mechanism by which EPA prevents certain diseases is its ability to decrease F_2_-IsoP generation [8].

### 3.2. IsoPs As Biomarkers of Lipid Peroxidation in Neurodegenerative Diseases

Oxidative stress is caused by an imbalance between free radicals production and antioxidant defenses in favor of the oxidation and leads to lipid peroxidation, membrane protein, and DNA damage and is thought to be important in the pathogenesis of a variety of neurological disorders, especially neurodegenerative diseases or atherosclerosis, cancer, and aging [42]. Lipid peroxidation is the most important source of free radical-mediated injury that directly damages neuronal membranes and yields a number of secondary products responsible for extensive cellular damage. Any specific repair process of lipid peroxidation does not exist as it does for proteins and DNA and this may explain why moderate levels of lipid peroxidation could have physiological significance for cell signaling and membrane remodeling [7]. One of the major targets of the lipid peroxidation process is the CNS. The brain is the most susceptible to oxidative damage because of the high oxygen consumption, the low levels of antioxidant enzymes (catalase and glutathione peroxidase), the elevated levels of iron (a potent catalyst for oxidant formation), and the ability to oxidize different substrates (e.g., membrane polyunsaturated fatty acids). Despite the fact that free radicals can attack many various critical biological molecules, such as DNA and cellular proteins, the high content of unsaturated lipids renders lipid peroxidation, the central feature of oxidant injury in the brain [43]. Peroxidation of membrane lipids affects neuronal homeostasis resulting in augmented membrane inflexibility, diminished activity of membrane-bound enzymes (e.g., sodium pump), destruction of membrane receptors, and changed permeability [44, 45]. One leading hypothesis is that the free radical-mediated oxidation of lipids contributes to the main pathological effects of oxidative stress in the brain. In support of this theory, increased levels of bioactive lipid peroxidation products have been identified in affected brain regions from humans with various neurodegenerative diseases [46, 47], as well as in corresponding animal models [48].

Due to the fact that free radicals are unstable and highly reactive, there are difficulties in direct measurement of their level. That is why elucidation of the importance of oxidative damage in neurological diseases is very hard. Because of their stability, the measurement of F_2_-IsoPs by mass spectrometry has been extensively employed as a marker of oxidant stress and is widely considered to be the gold-standard index of lipid peroxidation* in vivo* [49, 50]. IsoPs can be relatively easily quantified in body fluids because they are commonly found in urine, blood, and CSF and are also present in the exhaled air (Table 1). Their formation* in vivo* can be reliably monitored in every biological fluid by the noninvasive measurements of specific signals of lipid peroxidation, which tend to be sensitive and specific [51]. The measurement of F_2_-IsoPs has emerged as one of the most reliable approaches to assess oxidative stress status* in vivo*, providing an important tool to explore the role of oxidative stress in the pathogenesis of human disease. In the oxidative tissue injury the level of F_2_-IsoP is significantly increasing. The rapid development of analytical methods for IsoPs measurement helped clarify the role of the free radicals in human physiology and pathophysiology [52].

Measurement of F_4_-NPs, the stable product of free radical damage to DHA, also provides valuable data in exploring the role of oxidative stress in neurodegenerative diseases. The products of the IsoP pathway were found to have strong biological actions and therefore may participate as physiological mediators of the disease [59]. Research on brain-derived IsoPs has begun only a few years ago, but it has already provided convincing evidence on the usefulness of these markers in understanding the role of oxidative damage in brain diseases [60]. IsoPs as active products of free-radical-mediated peroxidation of AA contained in phospholipids of cell membranes and lipoproteins have a potential relevance to human neurodegenerative and demyelinating diseases. The role of free radical-induced oxidative damage in the pathogenesis of neurodegenerative disorders has been definitely established [61–65]. The elevated formation of F_2_-IsoPs has been observed in brain tissues and body fluids in numerous neurodegenerative diseases, including Alzheimer's disease [32], Parkinson's disease [6], Huntington's disease (HD) [66], Creutzfeldt-Jakob disease (CJD) [66], multiple sclerosis [55], and amyotrophic lateral sclerosis (ALS) [43].

The measurement of free F_2_-IsoPs in plasma or urine can be utilized to assess the endogenous formation of IsoPs but not to reveal the organ in which they are formed. Determining the levels of IsoPs in the unique fluid compartment—CSF, which reflects the ongoing metabolic activity of the brain, provides a great opportunity to reveal the occurrence of oxidative stress and lipid peroxidation in the brain [10, 44].

## 4. Multiple Sclerosis

Multiple sclerosis is a multifactorial, heterogeneous disease with several pathophysiological components: inflammation, demyelination, redox, axonal damage, and repair processes. These components are not uniformly contributed in patient populations but can individually predominate [67, 68]. MS is a leading cause of neurological disabilities in young adults and affects up to 2.4‰ of population in USA and Canada and up to 1.9‰ in some European countries. It is considered to be autoimmune, or at least its etiopathogenesis involves intensive autoaggressive immune response [69]. MS is heterogeneous disease on several grounds. There are several different clinical courses of this disorder. The most usual (over 80%) is relapsing-remitting course (RRMS) in which relapse occurs from time to time followed up by complete or partial recovery [67]. This stage of disease is characterized with multifocal inflammation, oedema, and cytokines actions. About half of RRMS patients after 10–20 years of disease lasting accumulate irreversible neurological deficits [67, 70]. This type of MS is known as secondary progressive (SPMS) that is dominated by neurodegeneration processes and progression of clinical symptoms [71]. The next 20% of MS patients with progressive symptoms from the onset have primary-progressive (PPMS) type. For the transition from RRMS to progressive stage axonal injury is responsible [67]. Neurodegeneration of demyelinated axons is a major cause of irreversible neurological disability in MS. Disability levels in progressive forms of MS patients often worsen despite a stable MRI T(2) (magnetic resonance) lesion burden [67]. The presence of oxidative stress in the absence of measurable inflammation could help explain this phenomenon [3, 47].

Currently classifications of biomarkers of MS are connected with the pathophysiological processes. It has been divided into seven categories:

1. alteration of the immune system (interleukins IL-1*β*, IL-2, IL-4, IL-6, IL-10, IL-12, IL-23, interferon (IFN*γ*), tumor necrosis factor (TNF*α*), transforming growth factor (TGF*β*), cytokines CXCR3/CXCL10—marker activated T cells; E-selectin, L-selectin, ICAM-1, VCAM-1, CD31, surface expression of LFA-1 and VLA-4 (adhesion molecules), CD40/CD40L, CD80, CD86, and heat shock proteins (hsp));
2. axonal/neuronal damage (Tau protein, 24S-hydroxycholesterol, N-acetylaspartic acid);
3. blood-brain barrier disruption (matrix metalloproteinases (MMPs): MMP-9 and their inhibitors (TIMP), platelet activating factor (PAF), and thrombomodulin);
4. demyelination (MBP and MBP-like material, proteolytic enzymes);
5. oxidative stress and excitotoxicity (nitric oxide derivatives, F_2_-IsoPs, and uric acid);
6. gliosis (glial fibrillary acid protein (GFAP), S-100 protein);
7. remyelination and repair (NCAM (neural cell adhesion molecule), CNTF (ciliary neurotrophic factor), MAP-2 + 13 (microtubule-associated protein-2 exon 13), and CPK-BB (creatine phosphatase BB) [67].

IsoPs are the candidate biomarkers of lipid peroxidation in MS. In diseases with a complex pathogenesis an individual biomarker is reflected in only one of many ongoing pathogenic processes [5, 67]. The data presented in our studies indicate that lipid peroxidation and oxidative stress in patients with MS may occur. It was found that the urine IsoPs level was over 6-fold elevated in patients with SPMS than in control [3]. The increased level of 15-F_2*t*_-IsoPs in CSF of MS patients has been described by Mattsson et al. [9]. To investigate the possible correlation between F_2_-IsoPs and the disease inflammatory activity it has been observed that the CSF levels of 15-F_2*t*_-IsoPs in patients with RRMS were not correlated with the clinical signs of the disease. These observations suggest that high levels of F_2_-IsoPs (about 9-fold higher than control subjects) may represent an index of degenerative phenomena, which persist also in the lack of an ongoing inflammatory activity. In other researches, Minghetti et al. [10] have found that the CSF level of the reliable marker of oxidative stress* in vivo*, 15-F_2*t*_-IsoP, is 3 times higher in patients with MS than in a benchmark group of subjects with other neurologic diseases. This increase was not correlated with the 15-F_2*t*_-IsoP levels and was much lower in steroid-treated patients. Clearly, the levels of 15-F_2*t*_-IsoP were associated with the degree of disability. What is more, in the spinal cord of mice during early progressive stages of experimental autoimmune encephalomyelitis (EAE) the elevated levels of F_2_-IsoPs and F_4_-NPs were observed [72, 73]. In white matter and myelin-forming oligodendrocytes the DHA levels are relatively low, which are affected in MS. So, F_2_-IsoPs might be preferable to F_4_-NPs as lipid peroxidation biomarkers in this demyelinating disease [66]. There are some studies which examine the correlation between levels of F_2_-IsoPs in CSF of MS patients, their healthy siblings, and unrelated controls. The CSF concentrations of F_2_-IsoPs in siblings of MS patients were significantly higher than in healthy controls. The F_2_-IsoPs levels in patients suffering from MS were intermediate between siblings, as well as controls. In patients with MS and siblings, the levels of F_2_-IsoPs were significantly correlated. These researches have been proved that siblings of MS patients have an increased oxidative stress response to the environmental and genetic factors that might be involved in MS pathogenesis [9].

## 5. Alzheimer's Disease

Alzheimer's disease (AD) is one of the major causes of dementia, which is characterized by the deposition of the amyloid *β* (A*β*) peptide and microtubule-associated protein tau in the brain [74, 75]. The critical role in the AD pathogenesis plays an abnormal tau phosphorylation. It has been proved that A*β* has capacity to interact with transition metals generating redox active ions, which precipitate in lipid peroxidation and cellular oxidative stress [76]. In other words, A*β* promotes cellular oxyradicals accumulation in neurons and glial cells in vulnerable regions of AD brain. Such oxidative stress may lead to many of the metabolic and neurodegenerative alterations observed in this disease [77]. Moreover, in tau phosphorylation, the mediation of oxidant toxicity by A*β* has been also implicated. Besides the oxidative stress, the mitochondrial dysfunction has been observed in AD [78]. A variety of markers of oxidative stress are increased, with a clear relationship with A*β* deposition and neurofibrillary degeneration has been observed in postmortem brain tissues from AD patients [79]. It has been reported that the activity and/or protein levels of several antioxidant enzymes were altered in AD brain regions, consistent with ongoing oxidative stress [11]. Increased F_2_-IsoPs and F_4_-NPs levels in the postmortem ventricular fluid from definite AD patients had been firstly demonstrated by Montine et al. [11]. The authors, given the partial overlap between CSF concentrations of F_2_-IsoPs in AD patients and healthy subjects, suggested that the quantification of CSF F_2_-IsoPs could not be utilized as an early marker of dementia. There was no correlation between CSF F_2_-IsoPs and age or duration of disease. This study concerns the relative small group of AD patients and probably may not be fully representative of the AD population [11]. In an independent study, Musiek and colleagues [80] demonstrated the formation of F_4_-NPs during peroxidation of DHA* in vitro *F_4_-NPs may be used as a marker of lipid peroxidation in the pathogenesis of neurodegenerative diseases, because in these diseases the elevated levels of F_4_-NPs is observed. Subsequently, they proved the presence of esterified F_4_-NPs in the human brain and showed abnormally high levels in occipital and temporal lobes of AD brains. Interestingly, while* in vitro* oxidation of DHA yields 3.4-fold higher levels of F_4_-NPs compared with F_2_-IsoPs, the CSF levels of these two classes of compounds showed a very close correlation in a small number of AD patients [66].

In Yao et al.'s [12] and Praticò et al.'s [13] researches, found that the contents of 15-F_2*t*_-IsoPs and IPF_2_alpha-VI were markedly elevated in the frontal and temporal lobes of AD brains compared to the corresponding cerebella and to the same regions of control brains. Moreover, there was also a significant correlation between the levels of the two IsoPs measured in each AD brain. In postmortem ventricular CSF, IPF_2_alpha-VI levels were higher in AD patients than in healthy people. In contrast, brains levels of 6-keto PGF_1_alpha, an index of prostaglandin production, and ventricular CSF 15-F_2*t*_-IsoP levels did not differ in AD and control subjects.

F_2_-IsoPs were measured also in plasma and in urine of AD patients. It has been shown that plasma and urinary levels were higher than controls, but only in the case of plasma the difference was statistically significant. So, plasma or urine content of IsoP in patients with AD reflects a specific increase in oxidative stress within the brain or a more generalized systemic oxidative stress remains to be determined. The authors also found that in the control group F_2_-IsoPs levels in females were higher than in males and suggested that this could be related to an increase in oxidative stress associated with the loss of estrogens in the postmenopausal period [81]. Indeed, estrogens can be antioxidants because of their phenolic structure [82] or may upregulate apolipoprotein E, favoring the formation of the apolipoprotein E/A-complex, and thus the sequestration of A*β*. Consistent with this hypothesis, Praticò et al. [13] demonstrated markedly elevated F_2_-IsoPs in the brains of aged apolipoprotein E-deficient mice compared with wild-type C5 [83].

## 6. Huntington's Disease

The abnormal expansions of an unstable cytosine-adenine-guanine repeat region at the 5′-end of a gene on chromosome 4 are the main cause of this disease. This genetic abnormality results in the expression of an expanded polyglutamine tract in huntingtin protein, which can aggregate in neuronal nuclei and dystrophic neuritis in Huntington's disease brains. The HD gene defect causing the death of specific populations of striatal neurons is still unknown. The elevated oxidative damage observed in areas of degeneration in patients' brains with HD and the increased free radical production in animal models indicate the involvement of oxidative stress either as a cause or as a consequence of the cell death cascade in the disease [66, 84]. There are a lot of studies suggesting that oxidative stress is prominent in the neostriatum of HD brains [85] and contributes to degeneration of the neostriatum. In patients suffering from HD, the mitochondrial dysfunction results in overproduction of ROS leading to oxidative and nitrosative stress [54, 86–88]. Such stress contributes to neuronal dysfunction by damaging the main structures: DNA, proteins, and lipids. It has been shown that the highly reactive product of nitric oxide and superoxide free radicals—peroxynitrite, which inhibits mitochondrial respiration and reduce antioxidant defenses in cells, is marked by increased of 3-nitrotyrosine (3-NT) levels [54, 87, 89]. The immunoreactivity of 3-NT is increased in postmortem HD brain tissue [85]. Also, increased levels of protein carbonyls in HD striatum and cerebral cortex have been observed [85]. It has been observed that 4-hydroxynonenal and malondialdehyde, lipid peroxidation products, are increased eightfold in HD human plasma [90] and also in postmortem brain tissue [84].

The measurement of the levels of F_2_-IsoPs in the CSF of HD patients indicates the contribution of oxidative stress to the pathogenesis of HD. The level of F_2_-IsoP in HD patients was significantly higher than in the control group. However, the overlap of levels between these groups suggested that the oxidative damage to the brain may not occur uniformly in the early phase of the disease. But like in AD, correlation between F_2_-IsoPs and age or disease duration there was not found; moreover no difference between men and women was observed [66].

In addition, in HD plasma the glutathione levels are significantly reduced [91]. Browne and Beal [14] suggest that in transgenic HD mice, there are increased immunostaining for malondialdehyde, 4-hydroxynonenal, and 15-F_2*t*_-IsoPs [54].

## 7. Creutzfeldt-Jakob Disease

Creutzfeldt-Jakob disease (CJD) is one of the most known human transmissible spongiform encephalopathies (TSEs) or prion diseases, a heterogeneous group of infectious, sporadic, and genetic disorders characterized by rapidly progressive dementia. The characteristic neuropathological hallmark of the disease is the amyloid deposition of the pathological form of a cellular protein (like in AD—A*β* or HD—huntingtin). The accumulation of the pathological prion protein is considered as a central event and is thought to trigger several pathogenetic mechanisms, eventually culminating in the typical spongiform degeneration [66].

The physiological functions of cellular prion protein are still unknown; however, due to its cooper binding ability it might play an important role in the oxidative homeostasis of the brain and could act as an antioxidant. These antioxidant properties may be related to its superoxide dismutase- (SOD-) like activity [15, 16]. Kralovicova et al. [15] have proved that these cells, which express higher levels of prion protein, are more resistant to oxidative stress. Wong et al. suggest [16] that the levels of several oxidative stress markers, protein carbonyl groups and products of lipid peroxidation, were increased in brain tissues of prion protein knockout mice [92]. In brains of mice infected with scrapie, the elevated levels of nitrotyrosine and heme oxygenase-1 had been found [93]. It has been suggested that the level of lipid peroxidation products is increased in brains of scrapie-infected mice and also prion proteins purified from brains of these animals possess a reduced SOD-like activity [94].

The increased levels of F_2_-IsoP in CSF of Creutzfeldt-Jakob patients have been observed in Minghetti et al.'s [17] researches. Also, another product of lipid peroxidation has been found to be unchanged in CSF from patients suffering from CJD in comparison to controls [95]. Arlt et al. [18] found that CSF lipids from patients suffering from CJD were more susceptible to oxidation process than those from nondemented controls. Thus, they observed that in the CJD patients, the levels of antioxidants and the amount of PUFAs were reduced. Their researches indicate that oxidative stress is elevated in CJD patients and the oxidative mechanisms are correlated with pathogenesis of this disease.

It has been observed that in patients with sporadic and familial CJD, CSF levels of 15-F_2*t*_-IsoP were about 2.5-fold higher than in patients with noninflammatory disorders. No correlation was found between 15-F_2*t*_-IsoPs and PGE_2_ and also 15-F_2*t*_-IsoP levels and age of patients nor polymorphism at codon 129 of the prion protein gene, indicating that lipid peroxidation and prostaglandin synthesis are unrelated phenomena in this disease. PGE_2_ concentrations, that were about 6.5-fold higher than in controls, were inversely correlated with patient survival; meanwhile, the levels of 15-F_2*t*_-IsoP were not correlated with the clinical duration of the disease. It has been suggested that the inflammation might be more relevant than oxidative stress to the pathogenesis of this particular disease [53, 66].

In other studies, it has been proved that the increased level of PGE_2_ in hippocampal is associated with a strong induction of COX-2 expression, which was elevated with progression of disease and is localized to microglial cells [56]. In sporadic CJD patients the shorter survival was associated with higher levels of PGE_2_ in CSF patients. PGE_2_ may be an index of disease severity rather than progression, because PGE_2_ levels were not dependent on the time of CSF sampling during the course of the disease [10]. PGE_2_ can be associated with neuronal death, because in neuroblastoma cells, prion proteins peptides increase PGE_2_ levels and COX-1 inhibitors protect against prion proteins toxicity [57]. Whether PGE_2_ contributes to neuronal death in CJD, is a consequence of neuronal apoptosis, or is just an index of the disease state remains to be established.

## 8. Amyotrophic Lateral Sclerosis

Amyotrophic lateral sclerosis (ALS) is a multifactorial and complex disease, in which genetic, environmental, or genetic-environmental interactions lead to motor neuronal degeneration. The deposition of a misfolded protein in neural tissue, in this instance copper/zinc SOD, is characteristic for the ALS and other neurodegenerative diseases [96, 97]. Several neuroinflammatory changes, such as increased levels of proinflammatory molecules, astrogliosis, and also microglial activation, which are characteristic for many neurodegenerative diseases, have been also found in spinal cord tissue from patients who died of ALS. These processes suggest that inflammation might promote motor neuron death. In addition, in ALS, high CSF levels of glutamate and excitotoxicity have been reported [98]. It has been proved that in ALS, oxidative stress is closely associated with motor neuron degeneration. Several recent clinical researches suggest that there exist a number of biomarkers for oxidative stress in ALS. Mitsumoto et al. [19] have observed that the level of urinary 15-F_2*t*_-IsoP and urinary 8-oxodG was higher among ALS patients than in control. No correlation has been found between age and urinary IsoPs. Protein carbonyl levels did not differ between patients suffering from ALS and controls, in contrast to urinary levels of IsoPs and urinary 8-oxodG, which are strongly correlated. This suggests that 15-F_2*t*_-IsoPs and 8-oxodG are biomarkers of oxidative stress in patients with ALS. [58, 99].

What is more, it has been proved that the well-established role of COX-2 in inflammation and in glutamate-dependent neurotoxicity is a basic hypothesis of COX-2 involvement in ALS pathogenesis. The increased COX-2 mRNA and protein were found in postmortem spinal cord of ALS patients. Together with the increase of PGE_2_ tissue levels, the elevated expression of COX-2 has been observed [100]. COX-2 is expressed in neurons in the spinal cord dorsal and ventral horns and also in dorsal root ganglia under normal conditions. The COX-2 expression was markedly evaluated and localized to both neurons and glial cells in postmortem spinal cord of ALS patients. It has been proved that COX-2 is associated with astrocytes and much lesser extent with glial cells [101]. Some studies suggest that inhibition of COX-2 may have therapeutic benefits by altering the cascade of events leading to the progressive neuronal death in ALS patients. But the efficacy of COX-2 inhibition in the presence of overt clinical signs of disease still remains unknown.

In addition, in spinal cords of sporadic ALS patients, the immunoreactivity of 15-deoxy-D12-14-PGJ_2_ (15d-PGJ_2_) has been found. 15d-PGJ_2_, this bioactive prostanoid produced by dehydration and isomerization of PGD_2_, activates the nuclear peroxisome proliferator—activated receptor *γ* (PPARy). PPARy is a critical transcription factor involved in adipocyte and monocyte differentiation.

This receptor can be considered as a potential therapeutic benefit of its activation in several inflammatory neurological diseases [31].

## Figures and Tables

**Table 1:** Isoprostanes as markers of oxidative stress in neurodegenerative diseases.

Classes of isoprostanes	Material	Disease	Study	Versus control	Reference
F2-IsoPs	CSF*, post mortem brain tissue, plasma, urinary	Alzheimer disease	*vivo *	High	[10–13, 32]
		Creutzfeldt-Jakob	*vivo*/*vitro *	High	[17, 18, 53]
		Huntington disease	*vivo *	High	[54]

8-iso PGF 2alfa	Urine	SPMS**	*vivo *	6-fold	[55]
	CSF	RRMS***	*vivo *	Higher	[9, 53, 56, 57]
		ALS****	*vivo *	Higher	[5, 19, 58]

CSF*: cerebrospinal fluid; SPMS**: secondary-progressive type of multiple sclerosis; RRMS***: relapsing-remitting type of multiple sclerosis. ALS****: amyotrophic lateral sclerosis.

## References

[1] Enciu AM, Gherghiceanu M, Popescu BO (2013). Triggers and effectors of oxidative stress at blood-brain barrier level: relevance for brain ageing and neurodegeneration. *Oxidative Medicine and Cellular Longevity*.

[2] Niki E (2008). Lipid peroxidation products as oxidative stress biomarkers. *BioFactors*.

[3] Santos R, Almodovar CR, Bulteau AL, Gomes CM (2013). Neurodegeneration, neurogenesis, and oxidative stress. *Oxidative Medicine and Cellular Longevity*.

[4] Cappellano G, Carecchio M, Fleetwood T (2013). Immunity and inflammation in neurodegenerative diseases. *American Journal of Neurodegenerative Disease*.

[5] Almer G, Teismann P, Stevic Z (2002). Increased levels of the pro-inflammatory prostaglandin PGE2 in CSF from ALS patients. *Neurology*.

[6] Farooqui T, Farooqui AA (2011). Lipid-mediated oxidative stress and inflammation in the pathogenesis of Parkinson’s disease. *Parkinson’s Disease*.

[7] Guest J, Grant R, Mori TA, Croft KD (2014). Changes in oxidative damage, inflammation and [NAD(H)] with age in cerebrospinal fluid. *PLoS ONE*.

[8] Roberts LJ, Milne GL (2009). Isoprostanes. *Journal of Lipid Research*.

[9] Mattsson N, Haghighi S, Andersen O (2007). Elevated cerebrospinal fluid F_2_-isoprostane levels indicating oxidative stress in healthy siblings of multiple sclerosis patients. *Neuroscience Letters*.

[10] Minghetti L, Greco A, Cardone F (2000). Increased brain synthesis of prostaglandin E_2_ and F_2_-isoprostane in human and experimental transmissible spongiform encephalopathies. *Journal of Neuropathology and Experimental Neurology*.

[11] Montine TJ, Quinn JF, Milatovic D (2002). Peripheral F_2_-isoprostanes and F_4_-neuroprostanes are not increased in Alzheimer’s disease. *Annals of Neurology*.

[12] Yao Y, Zhukareva V, Sung S (2003). Enhanced brain levels of 8,12-iso-iPF2*α*-VI differentiate AD from frontotemporal dementia. *Neurology*.

[13] Praticò D, Lee VMY, Trojanowski JQ, Rokach J, Fitzgerald GA (1998). Increased F_2_-isoprostanes in Alzheimer’s disease: evidence for enhanced lipid peroxidation *in vivo*. *FASEB Journal*.

[14] Browne SE, Beal MF (2006). Oxidative damage in Huntington’s disease pathogenesis. *Antioxidants and Redox Signaling*.

[15] Kralovicova S, Fontaine SN, Alderton A (2009). The effects of prion protein expression on metal metabolism. *Molecular and Cellular Neuroscience*.

[16] Wong BS, Pan T, Liu T (2000). Prion disease: a loss of antioxidant function?. *Biochemical and Biophysical Research Communications*.

[17] Minghetti L, Cardone F, Greco A (2002). Increased CSF levels of prostaglandin E_2_ in variant Creutzfeldt-Jakob disease. *Neurology*.

[18] Arlt S, Kontush A, Zerr I (2002). Increased lipid peroxidation in cerebrospinal fluid and plasma from patients with creutzfeldt-jakob disease. *Neurobiology of Disease*.

[19] Mitsumoto H, Santella R, Liu X (2008). Oxidative stress biomarkers in sporadic ALS. *Amyotrophic Lateral Sclerosis*.

[20] Zhang R, Zhang Q, Niu J (2014). Screening of microRNAs associated with Alzheimer's disease using oxidative stress cell model and different strains of senescence accelerated mice. *Journal of the Neurological Sciences*.

[21] Jacob KD, Noren N, Hooten N, Trzeciak AR, Evans MK (2013). Markers of oxidant stress that are clinically relevant in aging and age-related disease. *Mech Ageing*.

[22] Farooqui AA, Horrocks LA, Farooqui T (2007). Interactions between neural membrane glycerophospholipid and sphingolipid mediators: a recipe for neural cell survival or suicide. *Journal of Neuroscience Research*.

[23] Gonsette RE (2008). Neurodegeneration in multiple sclerosis: the role of oxidative stress and excitotoxicity. *Journal of the Neurological Sciences*.

[24] Halliwell B, Lee CYJ (2010). Using isoprostanes as biomarkers of oxidative stress: some rarely considered issues. *Antioxidants and Redox Signaling*.

[25] Barrera G (2012). Oxidative stress and lipid peroxidation products in cancer progression and therapy. *ISRN Oncology*.

[26] Dotan Y, Lichtenberg D, Pinchuk I (2004). Lipid peroxidation cannot be used as a universal criterion of oxidative stress. *Progress in Lipid Research*.

[27] Milne GL, Yin H, Hardy KD, Davies SS, Roberts LJ (2011). Isoprostane generation and function. *Chemical Reviews*.

[28] Roberts LJ, Morrow JD (2002). Products of the isoprostane pathway: unique bioactive compounds and markers of lipid peroxidation. *Cellular and Molecular Life Sciences*.

[29] Hardy KD, Cox BE, Milne GL, Yin H, Roberts LJ (2011). Nonenzymatic free radical-catalyzed generation of 15-deoxy-Δ^12,14^ J_2_-like compounds (deoxy-J_2_-isoprostanes) *in vivo*1. *Journal of Lipid Research*.

[30] Murphy RC, Fahy E (2010). Isoprostane nomenclature: more suggestions. *Prostaglandins Leukotrienes and Essential Fatty Acids*.

[31] Harris SG, Padilla J, Koumas L, Ray D, Phipps RP (2002). Prostaglandins as modulators of immunity. *Trends in Immunology*.

[32] Reich EE, Markesbery WR, Roberts LJ, Swift LL, Morrow JD, Montine TJ (2001). Brain regional quantification of F-ring and D-/E-ring isoprostanes and neuroprostanes in Alzheimer’s disease. *American Journal of Pathology*.

[33] Streck EL, Czapski GA, Gonçalves da Silva C (2013). Neurodegeneration, mitochondrial dysfunction, and oxidative stress. *Oxidative Medicine and Cellular Longevity*.

[34] Chen Y, Morrow JD, Roberts LJ (1999). Formation of reactive cyclopentenone compounds *in vivo* as products of the isoprostane pathway. *Journal of Biological Chemistry*.

[35] Chen Y, Zackert WE, Roberts LJ, Morrow JD (1999). Evidence for the formation of a novel cyclopentenone isoprostane, 15-A(2t)-isoprostane (8-iso-prostaglandin A2) *in vivo*. *Biochimica et Biophysica Acta: Molecular and Cell Biology of Lipids*.

[36] O’Brien RD (2009). Fats and oils analysis. *Fats and Oils: Formulating and Processing for Applications*.

[37] de Felice C, Signorini C, Durand T (2011). F_2_-dihomo-isoprostanes as potential early biomarkers of lipid oxidative damage in Rett syndrome. *Journal of Lipid Research*.

[38] Barden AE, Corcoran TB, Mas E (2012). Is there a role for isofurans and neuroprostanes in pre-eclampsia and normal pregnancy?. *Antioxidants and Redox Signaling*.

[39] Brooks JD, Milne GL, Yin H, Sanchez SC, Porter NA, Morrow JD (2008). Formation of highly reactive cyclopentenone isoprostane compounds (A 3/J3-isoprostanes) *in vivo* from eicosapentaenoic acid. *Journal of Biological Chemistry*.

[40] Comporti M, Signorini C, Arezzini B, Vecchio D, Monaco B, Gardi C (2008). F_2_-isoprostanes are not just markers of oxidative stress. *Free Radical Biology and Medicine*.

[41] Zaja-Milatovic S, Gupta RC, Aschner M, Milatovic D (2009). Protection of DFP-induced oxidative damage and neurodegeneration by antioxidants and NMDA receptor antagonist. *Toxicology and Applied Pharmacology*.

[42] Milatovic D, Montine TJ, Aschner M (2011). Measurement of isoprostanes as markers of oxidative stress. *Methods in Molecular Biology*.

[43] 'Amico ED, Factor-Litvak P, Santella RM, Mitsumoto H (2013). Clinical perspective on oxidative stress in sporadic amyotrophic lateral sclerosis. *Free Radical Biology & Medicine*.

[44] Wong-Ekkabut J, Xu Z, Triampo W, Tang IM, Tieleman DP, Monticelli L (2007). Effect of lipid peroxidation on the properties of lipid bilayers: a molecular dynamics study. *Biophysical Journal*.

[45] Yehuda S, Rabinovitz S, Carasso RL, Mostofsky DI (2002). The role of polyunsaturated fatty acids in restoring the aging neuronal membrane. *Neurobiology of Aging*.

[46] Montine TJ, Neely MD, Quinn JF (2002). Lipid peroxidation in aging brain and Alzheimer’s disease. *Free Radical Biology and Medicine*.

[47] Miller E, Walczak A, Saluk J, Ponczek MB, Majsterek I (2012). Oxidative modification of patient’s plasma proteins and its role in pathogenesis of multiple sclerosis. *Clinical Biochemistry*.

[48] Korade Z, Xu L, Mirnics K, Porter NA (2013). Lipid biomarkers of oxidative stress in a genetic mouse model of Smith-Lemli-Opitz syndrome. *Journal of Inherited Metabolic Disease*.

[49] Milne GL, Gao B, Terry ES, Zackert WE, Sanchez SC (2013). Measurement of F_2_-isoprostanes and isofurans using gas chromatography-mass spectrometry. *Free Radical Biology & Medicine*.

[50] Casetta B, Longini M, Proietti F, Perrone S, Buonocore G (2012). Development of a fast and simple LC-MS/MS method for measuring the F_2_-isoprostanes in newborns. *Journal of Maternal-Fetal and Neonatal Medicine*.

[51] Milne GL, Yin H, Morrow JD (2008). Human biochemistry of the isoprostane pathway. *Journal of Biological Chemistry*.

[52] Milne GL, Yin H, Hardy KD, Davies SS, Roberts LJ (2011). Isoprostane generation and function. *Chemical Reviews*.

[53] Bleich S, Kropp S, Degner D (2000). Creutzfeldt-Jakob disease and oxidative stress. *Acta Neurologica Scandinavica*.

[54] Montine TJ, Beal MF, Robertson D (1999). Cerebrospinal fluid F_2_-isoprostanes are elevated in Huntington’s disease. *Neurology*.

[55] Miller E, Mrowicka M, Saluk-Juszczak J, Ireneusz M (2011). The level of isoprostanes as a non-invasive marker for *in vivo* lipid peroxidation in secondary progressive multiple sclerosis. *Neurochemical Research*.

[56] Minghetti L, Cardone F, Greco A (2002). Increased CSF levels of prostaglandin E_2_ in variant Creutzfeldt-Jakob disease. *Neurology*.

[57] Walsh DT, Perry VH, Minghetti L (2000). Cyclooxygenase-2 is highly expressed in microglial-like cells in a murine model of prion disease. *Glia*.

[58] Consilvio C, Vincent AM, Feldman EL (2004). Neuroinflammation, COX-2, and ALS—a dual role?. *Experimental Neurology*.

[59] Roberts LJ, Fessel JP, Davies SS (2005). The biochemistry of the isoprostane, neuroprostane, and isofuran pathways of lipid peroxidation. *Brain Pathology*.

[60] Milatovic D, Aschner M (2009). Measurement of isoprostanes as markers of oxidative stress in neuronal tissue. *Current Protocols in Toxicology*.

[61] Melo A, Monteiro L, Lima RMF, de Oliveira DM, de Cerqueira MD, El-Bachá RS (2011). Oxidative stress in neurodegenerative diseases: mechanisms and therapeutic perspectives. *Oxidative Medicine and Cellular Longevity*.

[62] Ortiz GG, Pacheco-Moisés FP, Bitzer-Quintero OK (2014). Immunology and oxidative stress in multiple sclerosis: clinical and basic approach. *Journal of Cerebral Blood Flow & Metabolism*.

[63] Sultana R, Butterfield DA (2010). Role of oxidative stress in the progression of Alzheimer’s disease. *Journal of Alzheimer’s Disease*.

[64] Ayala-Peña S (2013). Role of oxidative DNA damage in mitochondrial dysfunction and Huntington's disease pathogenesis. *Free Radical Biology & Medicine*.

[65] Butterfield DA, Bader Lange ML, Sultana R (2010). Involvements of the lipid peroxidation product, HNE, in the pathogenesis and progression of Alzheimer’s disease. *Biochimica et Biophysica Acta: Molecular and Cell Biology of Lipids*.

[66] Greco A, Minghetti L, Levi G (2000). Isoprostanes, novel markers of oxidative injury, help understanding the pathogenesis of neurodegenerative diseases. *Neurochemical Research*.

[67] Miller E (2012). Neurodegenerative diseases. *Advances in Experimental Medicine and Biology*.

[68] Karussis D (2014). The diagnosis of multiple sclerosis and the various related demyelinating syndromes: a critical review. *Journal of Autoimmunity*.

[69] Gandhi S, Abramov AY (2012). Mechanism of oxidative stress in neurodegeneration. *Oxidative Medicine and Cellular Longevity*.

[70] Peterson LK, Fujinami RS (2007). Inflammation, demyelination, neurodegeneration and neuroprotection in the pathogenesis of multiple sclerosis. *Journal of Neuroimmunology*.

[71] Miller E, Mrowicka M, Malinowska K, Mrowicki J, Saluk-Juszczak J, Kȩdziora J (2011). Effects of whole-body cryotherapy on a total antioxidative status and activities of antioxidative enzymes in blood of depressive multiple sclerosis patients. *World Journal of Biological Psychiatry*.

[72] Ljubisavljevic S, Stojanovic I, Pavlovic D (2013). Suppression of the lipid peroxidation process in the CNS reduces neurological expression of experimentally induced autoimmune encephalomyelitis. *Folia Neuropathologica*.

[73] Greco A, Minghetti L (2004). Isoprostanes as biomarkers and mediators of oxidative injury in infant and adult central nervous system diseases. *Current Neurovascular Research*.

[74] Mondragón-Rodríguez S, Perry G, Zhu X, Boehm J (2012). Amyloid beta and tau proteins as therapeutic targets for Alzheimer’s disease treatment: rethinking the current strategy. *International Journal of Alzheimer’s Disease*.

[75] Savelieff MG, Lee S, Liu Y, Lim MH (2013). Untangling amyloid-*β*, tau, and metals in Alzheimer's disease. *ACS Chemical Biology*.

[76] Ellis G, Fang E, Maheshwari M (2010). Lipid oxidation and modification of amyloid-*β* (A*β*) *in vitro* and *in vivo*. *Journal of Alzheimer’s Disease*.

[77] Sultana R, Perluigi M, Butterfield AD (2013). Lipid peroxidation triggers neurodegeneration: a redox proteomics view into the Alzheimer disease brain. *Free Radical Biology & Medicine*.

[78] Pritchard SM, Dolan PJ, Vitkus A, Johnson GVW (2011). The toxicity of tau in Alzheimer disease: turnover, targets and potential therapeutics. *Journal of Cellular and Molecular Medicine*.

[79] Zhao Y, Zhao B (2013). Oxidative stress and the pathogenesis of Alzheimer's disease. *Oxidative Medicine and Cellular Longevity*.

[80] Musiek ES, Cha JK, Yin H (2004). Quantification of F-ring isoprostane-like compounds (F_4_-neuroprostanes) derived from docosahexaenoic acid *in vivo* in humans by a stable isotope dilution mass spectrometric assay. *Journal of Chromatography B: Analytical Technologies in the Biomedical and Life Sciences*.

[81] Long J, He P, Shen Y, Li R (2012). New evidence of mitochondria dysfunction in the female Alzheimer's disease brain: deficiency of estrogen receptor-*β*. *Journal of Alzheimer's Disease*.

[82] Mancini A, Raimondo S, Persano M (2013). Estrogens as antioxidant modulators in human fertility. *International Journal of Endocrinology*.

[83] Praticò D, Rokach J, Tangirala RK (1999). Brains of aged apolipoprotein E-deficient mice have increased levels of F_2_-isoprostanes, *in vivo* markers of lipid peroxidation. *Journal of Neurochemistry*.

[84] Sánchez-López F, Tasset I, Agüera E (2012). Oxidative stress and inflammation biomarkers in the blood of patients with Huntington's disease. *Neurological Research*.

[85] Sorolla MA, Reverter-Branchat G, Tamarit J, Ferrer I, Ros J, Cabiscol E (2008). Proteomic and oxidative stress analysis in human brain samples of Huntington disease. *Free Radical Biology and Medicine*.

[86] Browne SE, Beal MF (2006). Oxidative damage in Huntington’s disease pathogenesis. *Antioxidants and Redox Signaling*.

[87] Haun F, Nakamura T, Lipton SA (2013). Dysfunctional mitochondrial dynamics in the pathophysiology of neurodegenerative diseases. *Journal of Cell Death*.

[88] Stack EC, Matson WR, Ferrante RJ (2008). Evidence of oxidant damage in Huntington’s disease: translational strategies using antioxidants. *Annals of the New York Academy of Sciences*.

[89] Gan L, Johnson JA (2013). Oxidative damage and the Nrf2-ARE pathway in neurodegenerative diseases. *Biochimica et Biophysica Acta*.

[90] Carrizzo A, Di Pardo A, Maglione V (2014). Nitric oxide dysregulation in platelets from patients with advanced huntington disease. *PLoS ONE*.

[91] Stoy N, Mackay GM, Forrest CM (2005). Tryptophan metabolism and oxidative stress in patients with Huntington’s disease. *Journal of Neurochemistry*.

[92] Klepac N, Relja M, Klepac R, Hećimović S, Babić T, Trkulja V (2007). Oxidative stress parameters in plasma of Huntington’s disease patients, asymptomatic Huntington’s disease gene carriers and healthy subjects: a cross-sectional study. *Journal of Neurology*.

[93] Wong BS, Liu T, Li R (2001). Increased levels of oxidative stress markers detected in the brains of mice devoid of prion protein. *Journal of Neurochemistry*.

[94] Guentchev M, Voigtländer T, Haberler C, Groschup MH, Budka H (2000). Evidence for oxidative stress in experimental prion disease. *Neurobiology of Disease*.

[95] Wong BS, Brown DR, Pan T (2001). Oxidative impairment in scrapie-infected mice is associated with brain metals perturbations and altered antioxidant activities. *Journal of Neurochemistry*.

[96] Bate C, Rutherford S, Gravenor M, Reid S, Williams A (2002). Cyclo-oxygenase inhibitors protect against prion-induced neurotoxicity *in vitro*. *NeuroReport*.

[97] Boillée S, Vande Velde C, Cleveland D (2006). ALS: a disease of motor neurons and their nonneuronal neighbors. *Neuron*.

[98] Pradat PF, Attarian S, Camdessanché JP (2010). Research in amyotrophic lateral sclerosis: what is new in 2009?. *Revue Neurologique*.

[99] Mitsumoto H, Santella R, Liu X (2008). Oxidative stress biomarkers in sporadic ALS. *Amyotrophic Lateral Sclerosis*.

[100] Barber SC, Shaw PJ (2010). Oxidative stress in ALS: key role in motor neuron injury and therapeutic target. *Free Radical Biology and Medicine*.

[101] Almer G, Guegan C, Teismann P (2001). Increased expression of the pro-inflammatory enzyme cyclooxygenase-2 in amyotrophic lateral sclerosis. *Annals of Neurology*.

